# Phenyl bis­(morpholin-4-yl­amido)­phosphinate

**DOI:** 10.1107/S1600536811029734

**Published:** 2011-07-30

**Authors:** Mehrdad Pourayoubi, Hossein Eshtiagh-Hosseini, Monireh Negari, Marek Nečas

**Affiliations:** a Department of Chemistry, Ferdowsi University of Mashhad, Mashhad 91779, Iran; b Department of Chemistry, Faculty of Science, Masaryk University, Kotlarska 2, Brno CZ-61137, Czech Republic

## Abstract

In the title compound, C_14_H_23_N_4_O_4_P, the P atom is in a distorted tetra­hedral environment with bond angles in the range 96.87 (6)–119.86 (6)°. The two morpholinyl groups adopt a chair conformation. The phenyl ring is disordered over two sets of sites with equal occupancies [0.500 (2)]. In the crystal, adjacent mol­ecules are linked *via* N—H⋯O hydrogen bonds into an extended chain running parallel to the *a* axis. Only one of the amidate N—H groups is involved in hydrogen bonding.

## Related literature

For background to compounds having a P(=O)(O)(N)(N) skeleton, see: Sabbaghi *et al.* (2010 ▶). For bond lengths and angles in related structures, see: Ghadimi *et al.* (2009 ▶).
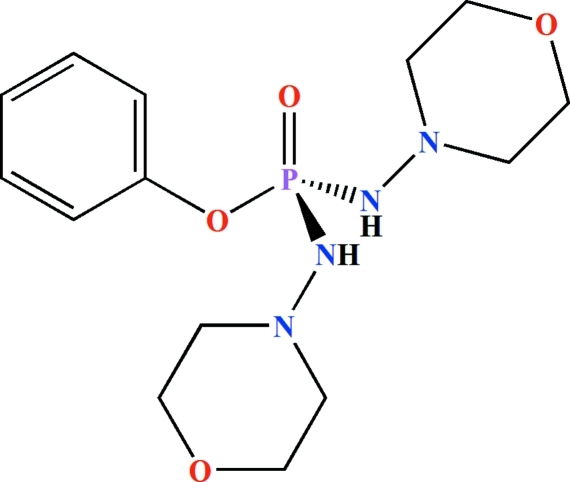


## Experimental

### 

#### Crystal data

C_14_H_23_N_4_O_4_P
*M* *_r_* = 342.33
Triclinic, 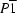

*a* = 4.7469 (2) Å
*b* = 12.3528 (5) Å
*c* = 14.2149 (5) Å
α = 90.542 (3)°
β = 98.389 (4)°
γ = 93.009 (4)°
*V* = 823.35 (6) Å^3^
*Z* = 2
Mo *K*α radiation
μ = 0.19 mm^−1^
*T* = 120 K
0.20 × 0.10 × 0.10 mm

#### Data collection

Oxford Diffraction Xcalibur S diffractometer
Absorption correction: multi-scan (*CrysAlis RED*; Oxford Diffraction, 2009 ▶) *T*_min_ = 0.877, *T*_max_ = 1.000
10043 measured reflections
2888 independent reflections
2272 reflections with *I* > 2σ(*I*)
*R*_int_ = 0.021

#### Refinement

*R*[*F* ^2^ > 2σ(*F* ^2^)] = 0.029
*wR*(*F* ^2^) = 0.070
*S* = 0.99
2888 reflections
262 parameters
162 restraints
H atoms treated by a mixture of independent and constrained refinement
Δρ_max_ = 0.22 e Å^−3^
Δρ_min_ = −0.32 e Å^−3^

### 

Data collection: *CrysAlis CCD* (Oxford Diffraction, 2009 ▶); cell refinement: *CrysAlis RED* (Oxford Diffraction, 2009 ▶); data reduction: *CrysAlis RED*; program(s) used to solve structure: *SHELXS97* (Sheldrick, 2008 ▶); program(s) used to refine structure: *SHELXL97* (Sheldrick, 2008 ▶); molecular graphics: *Mercury* (Macrae *et al.*, 2008 ▶); software used to prepare material for publication: *enCIFer* (Allen *et al.*, 2004 ▶).

## Supplementary Material

Crystal structure: contains datablock(s) I, global. DOI: 10.1107/S1600536811029734/gk2392sup1.cif

Structure factors: contains datablock(s) I. DOI: 10.1107/S1600536811029734/gk2392Isup2.hkl

Supplementary material file. DOI: 10.1107/S1600536811029734/gk2392Isup3.cml

Additional supplementary materials:  crystallographic information; 3D view; checkCIF report

## Figures and Tables

**Table 1:** Hydrogen-bond geometry (Å, °)

*D*—H⋯*A*	*D*—H	H⋯*A*	*D*⋯*A*	*D*—H⋯*A*
N3—H3*N*⋯O1^i^	0.823 (15)	2.093 (16)	2.8951 (16)	164.9 (15)

Symmetry code: (i) 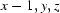.

**Fig. 1.**
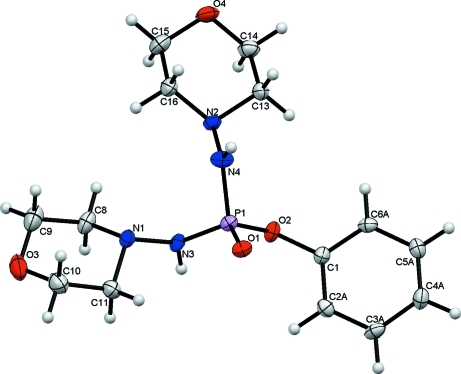
The molecular structure of the title compound with ellipsoids shown at the 50% probability level. The disorder is not shown.

**Fig. 2.**
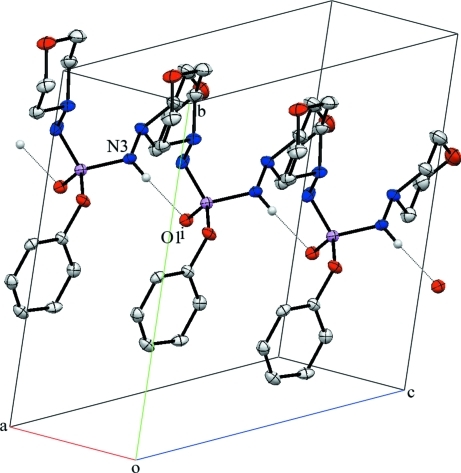
Partial packing view showing the formation of the chain through N—H···O hydrogen bond (shown as dotted lines). H atoms not involved in hydrogen bonding have been omitted for clarity. [Symmetry code: (i) x - 1, y, z]

## supplementary crystallographic information

### Comment

Structure determination of the title compound, P(O)[OC_6_H_5_][NHNC_4_H_8_O]_2_ (Fig. 1), was performed as a part of a project in our laboratory on the synthesis of compounds having a P(═O)(O)(N)(N) skeleton (Sabbaghi *et al.*, 2010). Single crystals of title compound were obtained from a mixture of CH_3_OH/CH_3_CN (4:1 *v*/*v*) after slow evaporation at room temperature.

The P═O (1.4705 (10) Å), P—O (1.5975 (10) Å) and P—N (1.6295 (13) Å & 1.6331 (13) Å) bond lengths and the C—O—P angle (122.17 (8)°) are standard for this category of compounds (Ghadimi *et al.*, 2009). The P atom has a distorted tetrahedral configuration (Fig. 1), the bond angles at the P atom vary in the range from 96.87 (6)° [the angle O2—P1—N3] to 119.86 (6)° [the angle O1—P1—N3].

In the crystal, the molecules are hydrogen-bonded in a linear arrangement parallel to [100] through N3—H3N···O1^i^ [symmetry code: (i) *x* - 1, *y*, *z*] hydrogen bond (Table 1, Fig. 2).

### Experimental

To a solution of phenyldichlorophosphate (2.507 mmol) in chloroform (15 ml), a solution of aminomorpholine (10.028 mmol) in chloroform (30 ml) was added at 273 K. After 4 h of stirring, the solvent was evaporated in vacuum. The solid was washed with distilled water. Single crystals, suitable for crystallography, were obtained from a solution of the title compound in methanol and acetonitrile (4:1) after slow evaporation at room temperature. IR (KBr, cm^-1^): 3278, 3107, 2945, 2869, 2830, 1588, 1488, 1223, 1107, 926, 869, 759, 698.

### Refinement

All carbon bound H atoms were placed at calculated positions and were refined as riding with their *U*_iso_ set to 1.2*U*_eq_ of the respective carrier atoms. Nitrogen bound H atoms were located in a difference Fourier map and refined isotropically. The disordered phenyl group was modeled over two sites using similarity restraints on anisotropic displacement parameters.

### Crystal data

**Table:**

C_14_H_23_N_4_O_4_P	*Z* = 2
*M**_r_* = 342.33	*F*(000) = 364
Triclinic, *P*1	*D*_x_ = 1.381 Mg m^−^^3^
Hall symbol: -P 1	Mo *K*α radiation, λ = 0.71073 Å
*a* = 4.7469 (2) Å	Cell parameters from 5042 reflections
*b* = 12.3528 (5) Å	θ = 3.3–27.2°
*c* = 14.2149 (5) Å	µ = 0.19 mm^−^^1^
α = 90.542 (3)°	*T* = 120 K
β = 98.389 (4)°	Prism, colorless
γ = 93.009 (4)°	0.20 × 0.10 × 0.10 mm
*V* = 823.35 (6) Å^3^	

### Data collection

**Table:**

Oxford Diffraction Xcalibur S diffractometer	2888 independent reflections
Radiation source: Enhance (Mo) X-ray Source	2272 reflections with *I* > 2σ(*I*)
graphite	*R*_int_ = 0.021
Detector resolution: 8.4353 pixels mm^-1^	θ_max_ = 25.0°, θ_min_ = 3.3°
ω scans	*h* = −5→5
Absorption correction: multi-scan (*CrysAlis RED*; Oxford Diffraction, 2009)	*k* = −14→14
*T*_min_ = 0.877, *T*_max_ = 1.000	*l* = −16→9
10043 measured reflections	

### Refinement

**Table:**

Refinement on *F*^2^	Primary atom site location: structure-invariant direct methods
Least-squares matrix: full	Secondary atom site location: difference Fourier map
*R*[*F*^2^ > 2σ(*F*^2^)] = 0.029	Hydrogen site location: inferred from neighbouring sites
*wR*(*F*^2^) = 0.070	H atoms treated by a mixture of independent and constrained refinement
*S* = 0.99	*w* = 1/[σ^2^(*F*_o_^2^) + (0.0404*P*)^2^*P*] where *P* = (*F*_o_^2^ + 2*F*_c_^2^)/3
2888 reflections	(Δ/σ)_max_ = 0.001
262 parameters	Δρ_max_ = 0.22 e Å^−^^3^
162 restraints	Δρ_min_ = −0.32 e Å^−^^3^

### Special details

**Table:**

Geometry. All e.s.d.'s (except the e.s.d. in the dihedral angle between two l.s. planes) are estimated using the full covariance matrix. The cell e.s.d.'s are taken into account individually in the estimation of e.s.d.'s in distances, angles and torsion angles; correlations between e.s.d.'s in cell parameters are only used when they are defined by crystal symmetry. An approximate (isotropic) treatment of cell e.s.d.'s is used for estimating e.s.d.'s involving l.s. planes.
Refinement. Refinement of *F*^2^ against ALL reflections. The weighted *R*-factor *wR* and goodness of fit *S* are based on *F*^2^, conventional *R*-factors *R* are based on *F*, with *F* set to zero for negative *F*^2^. The threshold expression of *F*^2^ > σ(*F*^2^) is used only for calculating *R*-factors(gt) *etc*. and is not relevant to the choice of reflections for refinement. *R*-factors based on *F*^2^ are statistically about twice as large as those based on *F*, and *R*- factors based on ALL data will be even larger.

### Fractional atomic coordinates and isotropic or equivalent isotropic displacement parameters (Å^2^)

**Table:**

	*x*	*y*	*z*	*U*_iso_*/*U*_eq_	Occ. (<1)
P1	0.34183 (7)	0.63069 (3)	0.29935 (3)	0.01615 (12)	
O1	0.55619 (19)	0.55524 (7)	0.33888 (7)	0.0196 (2)	
O2	0.11776 (19)	0.58245 (7)	0.21233 (7)	0.0196 (3)	
O3	0.1822 (2)	0.80213 (10)	0.63761 (8)	0.0382 (3)	
O4	0.3795 (2)	1.01932 (8)	0.11186 (8)	0.0310 (3)	
N3	0.1088 (3)	0.67116 (10)	0.36409 (9)	0.0180 (3)	
C1	0.1500 (3)	0.48228 (11)	0.16832 (11)	0.0169 (3)	
C2A	0.1144 (7)	0.3882 (2)	0.2144 (3)	0.0201 (8)	0.500 (2)
H2AA	0.0754	0.3896	0.2781	0.024*	0.500 (2)
C3A	0.1348 (8)	0.2902 (2)	0.1688 (3)	0.0228 (8)	0.500 (2)
H3AA	0.1112	0.2236	0.2004	0.027*	0.500 (2)
C4A	0.192 (5)	0.2917 (13)	0.0735 (11)	0.0236 (19)	0.500 (2)
H4AA	0.2198	0.2253	0.0426	0.028*	0.500 (2)
C5A	0.2073 (6)	0.3829 (2)	0.0270 (2)	0.0222 (8)	0.500 (2)
H5AA	0.2332	0.3813	−0.0381	0.027*	0.500 (2)
C6A	0.1858 (6)	0.4815 (2)	0.0724 (2)	0.0193 (8)	0.500 (2)
H6AA	0.1953	0.5475	0.0388	0.023*	0.500 (2)
C2B	−0.0552 (7)	0.4023 (2)	0.1721 (2)	0.0220 (8)	0.500 (2)
H2BA	−0.2160	0.4145	0.2028	0.026*	0.500 (2)
C3B	−0.0253 (8)	0.3024 (3)	0.1303 (3)	0.0276 (9)	0.500 (2)
H3BA	−0.1718	0.2470	0.1306	0.033*	0.500 (2)
C4B	0.203 (5)	0.2816 (13)	0.0897 (11)	0.023 (2)	0.500 (2)
H4BA	0.2230	0.2116	0.0642	0.027*	0.500 (2)
C5B	0.4184 (6)	0.3673 (2)	0.0852 (2)	0.0255 (9)	0.500 (2)
H5BA	0.5776	0.3553	0.0538	0.031*	0.500 (2)
C6B	0.3922 (6)	0.4675 (2)	0.1271 (2)	0.0214 (8)	0.500 (2)
H6BA	0.5358	0.5241	0.1274	0.026*	0.500 (2)
N1	0.2242 (2)	0.72689 (9)	0.45092 (9)	0.0187 (3)	
C8	0.0377 (3)	0.81391 (12)	0.46689 (12)	0.0274 (4)	
H8A	−0.1571	0.7832	0.4706	0.033*	
H8B	0.0268	0.8651	0.4134	0.033*	
C9	0.1576 (4)	0.87271 (13)	0.55883 (13)	0.0374 (5)	
H9A	0.3480	0.9064	0.5530	0.045*	
H9B	0.0319	0.9314	0.5703	0.045*	
C10	0.3616 (4)	0.71670 (14)	0.62119 (12)	0.0330 (4)	
H10A	0.3772	0.6673	0.6759	0.040*	
H10B	0.5552	0.7478	0.6160	0.040*	
C11	0.2460 (3)	0.65330 (12)	0.53168 (11)	0.0257 (4)	
H11A	0.3746	0.5950	0.5217	0.031*	
H11B	0.0557	0.6194	0.5373	0.031*	
N4	0.5044 (3)	0.73922 (10)	0.26441 (10)	0.0217 (3)	
N2	0.3561 (2)	0.82518 (9)	0.21815 (9)	0.0182 (3)	
C13	0.3853 (3)	0.82364 (12)	0.11680 (11)	0.0219 (4)	
H13A	0.2914	0.7562	0.0862	0.026*	
H13B	0.5898	0.8251	0.1096	0.026*	
C14	0.2503 (3)	0.92070 (12)	0.06897 (12)	0.0280 (4)	
H14A	0.2714	0.9192	0.0007	0.034*	
H14B	0.0440	0.9171	0.0737	0.034*	
C15	0.3467 (4)	1.02243 (12)	0.20977 (13)	0.0334 (4)	
H15A	0.1412	1.0206	0.2156	0.040*	
H15B	0.4364	1.0911	0.2393	0.040*	
C16	0.4816 (3)	0.92776 (12)	0.26208 (12)	0.0267 (4)	
H16A	0.6896	0.9322	0.2603	0.032*	
H16B	0.4514	0.9308	0.3295	0.032*	
H3N	−0.035 (3)	0.6311 (12)	0.3652 (11)	0.024 (4)*	
H4N	0.674 (4)	0.7378 (13)	0.2627 (12)	0.032 (5)*	

### Atomic displacement parameters (Å^2^)

**Table:**

	*U*^11^	*U*^22^	*U*^33^	*U*^12^	*U*^13^	*U*^23^
P1	0.01465 (19)	0.01504 (19)	0.0181 (3)	0.00138 (14)	−0.00015 (16)	0.00092 (16)
O1	0.0163 (5)	0.0179 (5)	0.0238 (7)	0.0023 (4)	−0.0004 (4)	0.0032 (5)
O2	0.0187 (5)	0.0187 (5)	0.0201 (7)	0.0048 (4)	−0.0027 (5)	−0.0049 (5)
O3	0.0424 (7)	0.0456 (7)	0.0272 (8)	−0.0048 (6)	0.0110 (6)	−0.0135 (6)
O4	0.0410 (7)	0.0185 (6)	0.0328 (8)	−0.0035 (5)	0.0047 (6)	0.0074 (5)
N3	0.0157 (6)	0.0174 (6)	0.0196 (8)	−0.0032 (5)	−0.0004 (6)	−0.0047 (6)
C1	0.0163 (7)	0.0169 (7)	0.0168 (9)	0.0032 (6)	−0.0004 (6)	−0.0007 (7)
C2A	0.0189 (16)	0.0240 (16)	0.0173 (19)	0.0026 (13)	0.0012 (15)	0.0018 (14)
C3A	0.0257 (18)	0.0142 (15)	0.026 (2)	−0.0001 (14)	−0.0026 (18)	0.0050 (15)
C4A	0.028 (3)	0.023 (4)	0.020 (4)	0.006 (3)	0.000 (4)	−0.005 (3)
C5A	0.0229 (17)	0.0264 (17)	0.0173 (19)	0.0017 (13)	0.0037 (15)	−0.0025 (14)
C6A	0.0189 (16)	0.0177 (14)	0.0203 (19)	−0.0018 (12)	0.0000 (15)	0.0051 (13)
C2B	0.0207 (17)	0.0256 (16)	0.020 (2)	0.0007 (13)	0.0047 (16)	0.0038 (14)
C3B	0.0274 (19)	0.0203 (16)	0.033 (2)	−0.0035 (15)	0.0000 (18)	0.0058 (16)
C4B	0.033 (3)	0.012 (2)	0.022 (5)	0.009 (2)	−0.003 (4)	0.005 (3)
C5B	0.0223 (16)	0.0326 (17)	0.022 (2)	0.0087 (13)	0.0020 (15)	−0.0064 (15)
C6B	0.0180 (16)	0.0232 (15)	0.022 (2)	0.0006 (12)	0.0003 (14)	0.0008 (14)
N1	0.0203 (6)	0.0183 (6)	0.0169 (8)	0.0005 (5)	0.0007 (6)	−0.0030 (6)
C8	0.0283 (8)	0.0213 (8)	0.0322 (11)	0.0036 (7)	0.0028 (8)	−0.0077 (7)
C9	0.0410 (10)	0.0298 (9)	0.0412 (13)	0.0025 (8)	0.0063 (9)	−0.0134 (9)
C10	0.0393 (10)	0.0366 (10)	0.0228 (11)	−0.0037 (8)	0.0059 (8)	−0.0001 (8)
C11	0.0318 (9)	0.0248 (8)	0.0207 (10)	−0.0010 (7)	0.0052 (8)	0.0026 (8)
N4	0.0140 (6)	0.0227 (7)	0.0279 (9)	0.0020 (5)	0.0007 (6)	0.0088 (6)
N2	0.0222 (6)	0.0142 (6)	0.0172 (8)	0.0013 (5)	−0.0011 (6)	0.0035 (6)
C13	0.0272 (8)	0.0201 (8)	0.0177 (10)	−0.0015 (6)	0.0022 (7)	−0.0006 (7)
C14	0.0341 (9)	0.0240 (8)	0.0241 (11)	−0.0027 (7)	−0.0006 (8)	0.0055 (7)
C15	0.0449 (10)	0.0179 (8)	0.0381 (12)	−0.0018 (7)	0.0097 (9)	−0.0020 (8)
C16	0.0345 (9)	0.0223 (8)	0.0224 (10)	−0.0063 (7)	0.0040 (8)	−0.0034 (7)

### Geometric parameters (Å, °)

**Table:**

P1—O1	1.4705 (10)	C5B—C6B	1.387 (4)
P1—O2	1.5975 (10)	C5B—H5BA	0.9500
P1—N4	1.6295 (13)	C6B—H6BA	0.9500
P1—N3	1.6331 (13)	N1—C8	1.4645 (17)
O2—C1	1.4068 (16)	N1—C11	1.4663 (19)
O3—C9	1.421 (2)	C8—C9	1.510 (2)
O3—C10	1.4286 (19)	C8—H8A	0.9900
O4—C15	1.4233 (19)	C8—H8B	0.9900
O4—C14	1.4249 (17)	C9—H9A	0.9900
N3—N1	1.4297 (17)	C9—H9B	0.9900
N3—H3N	0.823 (15)	C10—C11	1.505 (2)
C1—C2A	1.354 (3)	C10—H10A	0.9900
C1—C2B	1.358 (3)	C10—H10B	0.9900
C1—C6B	1.384 (3)	C11—H11A	0.9900
C1—C6A	1.399 (3)	C11—H11B	0.9900
C2A—C3A	1.382 (4)	N4—N2	1.4186 (16)
C2A—H2AA	0.9500	N4—H4N	0.811 (16)
C3A—C4A	1.421 (17)	N2—C16	1.4656 (18)
C3A—H3AA	0.9500	N2—C13	1.4674 (18)
C4A—C5A	1.316 (15)	C13—C14	1.510 (2)
C4A—H4AA	0.9500	C13—H13A	0.9900
C5A—C6A	1.389 (4)	C13—H13B	0.9900
C5A—H5AA	0.9500	C14—H14A	0.9900
C6A—H6AA	0.9500	C14—H14B	0.9900
C2B—C3B	1.388 (4)	C15—C16	1.512 (2)
C2B—H2BA	0.9500	C15—H15A	0.9900
C3B—C4B	1.33 (2)	C15—H15B	0.9900
C3B—H3BA	0.9500	C16—H16A	0.9900
C4B—C5B	1.44 (2)	C16—H16B	0.9900
C4B—H4BA	0.9500		
			
O1—P1—O2	114.63 (5)	N1—C8—H8A	109.9
O1—P1—N4	108.95 (6)	C9—C8—H8A	109.9
O2—P1—N4	108.62 (6)	N1—C8—H8B	109.9
O1—P1—N3	119.86 (6)	C9—C8—H8B	109.9
O2—P1—N3	96.87 (6)	H8A—C8—H8B	108.3
N4—P1—N3	106.95 (7)	O3—C9—C8	112.07 (14)
C1—O2—P1	122.17 (8)	O3—C9—H9A	109.2
C9—O3—C10	109.51 (13)	C8—C9—H9A	109.2
C15—O4—C14	109.64 (12)	O3—C9—H9B	109.2
N1—N3—P1	115.73 (9)	C8—C9—H9B	109.2
N1—N3—H3N	116.7 (11)	H9A—C9—H9B	107.9
P1—N3—H3N	116.9 (11)	O3—C10—C11	111.43 (13)
C2A—C1—C6B	103.2 (2)	O3—C10—H10A	109.3
C2B—C1—C6B	122.9 (2)	C11—C10—H10A	109.3
C2A—C1—C6A	120.6 (2)	O3—C10—H10B	109.3
C2B—C1—C6A	103.1 (2)	C11—C10—H10B	109.3
C2A—C1—O2	120.66 (18)	H10A—C10—H10B	108.0
C2B—C1—O2	117.71 (18)	N1—C11—C10	109.01 (13)
C6B—C1—O2	119.37 (17)	N1—C11—H11A	109.9
C6A—C1—O2	118.26 (16)	C10—C11—H11A	109.9
C1—C2A—C3A	120.0 (3)	N1—C11—H11B	109.9
C1—C2A—H2AA	120.0	C10—C11—H11B	109.9
C3A—C2A—H2AA	120.0	H11A—C11—H11B	108.3
C2A—C3A—C4A	118.2 (7)	N2—N4—P1	122.73 (9)
C2A—C3A—H3AA	120.9	N2—N4—H4N	118.0 (12)
C4A—C3A—H3AA	120.9	P1—N4—H4N	117.8 (12)
C5A—C4A—C3A	121.4 (13)	N4—N2—C16	108.27 (11)
C5A—C4A—H4AA	119.3	N4—N2—C13	109.67 (11)
C3A—C4A—H4AA	119.3	C16—N2—C13	109.55 (11)
C4A—C5A—C6A	120.4 (8)	N2—C13—C14	109.89 (12)
C4A—C5A—H5AA	119.8	N2—C13—H13A	109.7
C6A—C5A—H5AA	119.8	C14—C13—H13A	109.7
C5A—C6A—C1	119.0 (3)	N2—C13—H13B	109.7
C5A—C6A—H6AA	120.5	C14—C13—H13B	109.7
C1—C6A—H6AA	120.5	H13A—C13—H13B	108.2
C1—C2B—C3B	118.6 (3)	O4—C14—C13	111.09 (12)
C1—C2B—H2BA	120.7	O4—C14—H14A	109.4
C3B—C2B—H2BA	120.7	C13—C14—H14A	109.4
C4B—C3B—C2B	122.1 (8)	O4—C14—H14B	109.4
C4B—C3B—H3BA	119.0	C13—C14—H14B	109.4
C2B—C3B—H3BA	119.0	H14A—C14—H14B	108.0
C3B—C4B—C5B	118.9 (13)	O4—C15—C16	111.29 (13)
C3B—C4B—H4BA	120.6	O4—C15—H15A	109.4
C5B—C4B—H4BA	120.6	C16—C15—H15A	109.4
C6B—C5B—C4B	119.7 (9)	O4—C15—H15B	109.4
C6B—C5B—H5BA	120.2	C16—C15—H15B	109.4
C4B—C5B—H5BA	120.2	H15A—C15—H15B	108.0
C1—C6B—C5B	117.8 (3)	N2—C16—C15	110.24 (13)
C1—C6B—H6BA	121.1	N2—C16—H16A	109.6
C5B—C6B—H6BA	121.1	C15—C16—H16A	109.6
N3—N1—C8	108.48 (11)	N2—C16—H16B	109.6
N3—N1—C11	111.34 (11)	C15—C16—H16B	109.6
C8—N1—C11	109.63 (13)	H16A—C16—H16B	108.1
N1—C8—C9	108.73 (13)		

### Hydrogen-bond geometry (Å, °)

**Table:**

*D*—H···*A*	*D*—H	H···*A*	*D*···*A*	*D*—H···*A*
N3—H3N···O1^i^	0.823 (15)	2.093 (16)	2.8951 (16)	164.9 (15)

Symmetry codes: (i) *x*−1, *y*, *z*.
